# Social Cognition and Oxytocin in Huntington’s Disease: New Insights

**DOI:** 10.3390/brainsci8090161

**Published:** 2018-08-26

**Authors:** Elisa Unti, Sonia Mazzucchi, Daniela Frosini, Cristina Pagni, Gloria Tognoni, Lionella Palego, Laura Betti, Fabiana Miraglia, Gino Giannaccini, Roberto Ceravolo

**Affiliations:** 1 Neurology Unit, Apuane Hospital, 54100 Massa-Carrara, Italy; 2 Department of Clinical and Experimental Medicine, University of Pisa, 56126 Pisa, Italy; mazzucchi.s@gmail.com (S.M.); danielafrosini80@gmail.com (D.F.); cri_pagni@yahoo.it (C.P.); g.tognons@gmail.com (G.T.); lionella.palego@unipi.it (L.P.); roberto.ceravolo@med.unipi.it (R.C.); 3 Department of Pharmacy, University of Pisa, 56126 Pisa, Italy; laura.betti@unipi.it (L.B.); miragliafabiana@gmail.com (F.M.); gino.giannaccini@unipi.it (G.G.)

**Keywords:** Huntington’s Disease, oxytocin plasma levels, social cognition

## Abstract

This study is aimed at relating social cognition in Huntington’s Disease (HD) to plasma levels of the social hormone oxytocin (OT). Indeed, HD patients commonly display reduced social skills and OT is involved in bonding behavior and improved recognition of facial emotions. Twelve mild-symptomatic HD patients (stage II Shoulson & Fahn) and 11 gender/age matched controls (healthy controls, HC), without concurrent psychiatric disorders, were investigated at baseline (T_0_) for OT plasma levels and social cognition through an extensive battery of neuropsychological tests. Social cognition was also re-examined after two years (T1) in 8 of the 12 patients. Results showed a trend for reduced T_0_-OT levels in HD vs. HC, mean ± stardard deviation: 6.5 ± 2.4 vs. 9.9 ± 7.2 pg/mL, without reaching statistical significance. At T_0_, patients showed significantly lower performances than controls at the “Faux-Pas” and “Strange Stories” tests (*p* < 0.05; *p* < 0.01); a reduced perception of visual emotions (*p* < 0.01) and verbal stimuli (*p* < 0.01) was also reported, involving anger, fear, and sadness (*p* < 0.05; *p* < 0.01). Additionally, in the HD population, OT concentrations positively correlated with T1-performances at Neutral\Faux-Pas test (*p* < 0.05), whereas the cognitive Montreal Cognitive Assessment (MoCA) and Mini Mental State Examination (MMSE) scores positively correlated with psychosocial perception at the “Strange Stories” and Karolinska Directed Emotional Faces (KDEF) tests (*p* < 0.05). This study, despite its limitations, supports correlations between OT and HD social cognition, suggesting a possible therapeutic use of this hormone. More subjects and additional body tissues/fluids, such as cerebrospinal fluid, should be investigated to confirm this hypothesis.

## 1. Introduction

Huntington’s Disease (HD) is a rare monogenic autosomal dominant disease characterized by psychiatric, cognitive, and motor symptoms [1].

The clinical diagnosis of HD currently requires the presence of motor symptoms, mainly chorea; however, non-motor symptoms, such as psychiatric and cognitive disturbances, can manifest several years before [1].

Neurocognitive impairment in HD can be assessed with traditional neuropsychological batteries, exploring memory and executive functioning [2,3], but also by investigating impairment of social cognition [4]. Social cognition represents a cognitive domain that embraces different themes, including the perception of facial expression, prosody, and the “Theory of Mind” (ToM).

One aspect of social cognition, the recognition of emotion from faces, has been investigated in HD patients in a relatively large number of studies within the last 20 years, with particular attention to negative emotions [4,5,6]. Moreover, impaired emotion recognition has been evidenced even in response to stimuli other than faces (vocal, body language) [7,8].

The ToM is the ability to attribute mental states (feelings, beliefs, intentions, and desires) to others, and to understand and predict others’ behavior based on their mental states [9]. The ToM is not an entirely homogeneous concept, and some authors have suggested that ToM includes both affective and cognitive components, involved in reasoning versus decoding of mental states in HD patients [10].

In 1985, the striatum was identified as the main structure involved in the neuropathological process in HD [11]; however, in the attempt to explain cognitive–behavioral problems and non-motor aspects of the disease, such as excessive sweating, hyperphagia, weight loss, sexual disorders, and autonomic disorders, the attention was shifted to other areas of the central nervous system (CNS), and in particular the hypothalamus.

In this regard, a recent postmortem study has shown significant neuronal loss of the lateral tuberal nucleus and paraventricular nucleus, and a reduction of orexin, oxytocin (OT), and vasopressin-expressing neurons, with increased positivity for the transcript of cocaine–amphetamine and amine regulation [12].

OT is a nonapeptide known to play a pivotal role in a variety of complex social behaviors [13]. Two populations of neurons are responsible for the synthesis of OT: the magnocellular neurons of the supraoptic and paraventricular nuclei, which release the hormone mainly in the hypothalamic–hypophysis circle, and the parvocellular neurons located in the dorsal portion of the paraventricular nucleus that release OT from their axons at the level of limbic areas, such as the amygdala, bed nucleus of the stria terminalis, lateral septum, locus coeruleus, and hippocampal and mesencephalic areas [14].

Initial animal and human research mostly focused on exploring the function of OT in childbirth lactation and mother–child bonding [15,16,17,18]. More recently, an increasing amount of evidence supported the involvement of OT in promoting prosocial behavior [19], interpersonal bonding [20], and trust in adult relationships [21].

The relationship between OT and social cognition is supported by impairment of social recognition, which is associated with learning and memory integrity, in oxytocin-knockout mice [22]. Normal functioning can be restored by the administration of OT in the amygdala in particular [23]. The role of OT in social cognition has been supported even in a placebo-controlled crossover study conducted on HD patients versus controls. In this study, after the administration of 24 international units (UI) of intranasal OT, the pattern of activation to disgust stimuli was normalized in the HD group to similar levels as in the controls [24].

On the basis of the aforementioned data, we aimed to investigate, through an extensive and targeted battery of neuropsychological tests, the actual impairment of social cognition in patients affected by HD. A secondary objective of the study was to understand the role of OT in HD, and the relationship between the blood levels of this hormone and social cognition.

## 2. Material and Methods

### 2.1. Study Population

Twelve patients (4 females, 8 males) and 11 controls (3 females, 8 males) were enrolled in this study. These same subjects had also been recruited for a concomitant study conducted on platelet brain derived neurotrophic factor (BDNF) and serotonin transporter (SERT) [25]. Accordingly, all subjects introduced in this study were enrolled at the Ambulatory care of the Neurology section, Department of Clinical and Experimental Medicine, University of Pisa. Baseline visits included clinical evaluation, performed using the Unified Huntington’s Disease Rating Scale (UHDRS by the Huntington Study Group) [26], blood sampling for OT, and neuropsychological evaluation. Inclusion criteria were: Age > 40 years, and diagnosis of HD by means of the UHDRS scale, confirmed by genetic tests.

None of the subjects showed alterations of activity of daily living nor fulfilled the Diagnostic and Statistical Manual of Mental Disorders (DSM-V) criteria for dementia [27].

Patients present CAG pathologic expansion 40.5 ± 2.5 and UHDRS motor score at the baseline 36.6 ± 8.9 and disease duration 69 ± 5.7, HD-stage II [28].

Exclusion criteria were: age ≤ 40 years, presence of metabolic illnesses, and alcohol or drug abuse. Healthy Control subjects (HC) were recruited among outpatients of the Ambulatory care of the Neurology section, Department of Clinical and Experimental Medicine, University of Pisa, recovering from mild neurological disorders not affecting CNS. Recruitment was carried out by skilled neurologists from the Department. To avoid gender-dependent hormone interference, all women were recruited under an untreated postmenopausal phase. After fulfilling inclusion criteria, patients and controls gave written informed consent to participate in the study. A short-term wash-out period (7 days) from antidepressants and/or neuroleptics was applied to all HD patients before the beginning of the study and baseline examination (T0).

After two years (T1) 8 out of 11 patients were re-examined, using the same sociocognitive and neuropsychological tests. At the follow-up (T1), OT plasma level examinations were not performed due to patients’ refusal.

### 2.2. Measurement of Oxytocin Plasma Levels

#### Chemicals

All reagents and chemicals employed for this study were of the best analytical grade.

### 2.3. Blood Collection and Plasma Extraction

Basal levels of OT were appraised in 12-h-fasted patients and controls. 

Patients underwent a drug “wash-out” period of 7 days before starting the investigation, according to guidelines from the Ethical Committee of the University of Pisa (Ethical Approval Code: 431/2014).

Briefly, 15 mL of peripheral venous blood was withdrawn between 8 and 9 a.m. using vacutainer tubes, containing 1 mg/mL EDTA. Blood was gently mixed, immediately transferred into Falcon tubes containing 0.1 mg/mL Aprotinin, and centrifuged as described previously [25]. A vacuum manifold cartridge device and Oasis C18 cartridges (Waters Spa, Milan, Italy) were used for the OT extraction procedure. To improve the method’s sensitivity, the OT solid-phase extraction from plasma samples was optimized according to [29] and carried out as follows: after the blood centrifugation steps, the ensuing plasma was carefully measured for its volume and transferred into Corex glass tubes, acidified 1:1 (*v:v*) with 0.1 M HCl, and subsequently centrifuged at 48,000× *g* for 15 min at 4 °C; the resulting supernatant was measured again and loaded on Oasis column, previously activated by 10 mL methanol and 20 mL H_2_O (HPLC gradient grade, Simplicity, Millipore, Italy); a subsequent washing step with 10 mL of 4% acetic acid was applied to eliminate additional contaminants; OT was eluted from C18 cartridges using 2 mL of methanol. The final eluting volume was dried under high vacuum, using an SC100 SpeedVac centrifuge (Savant, Rome, Italy), connected to a condensation vapor RT4104 trap, preset at −80 °C (Savant, Rome, Italy). Dried samples were stored at −80 °C until assay.

### 2.4. Plasma Oxytocin Enzyme-Immunoassay (EIA)

To measure OT levels in plasma samples from patients and controls, a competitive and colorimetric 96-well microplate Enzyme–Immunoassay (EIA) (Enzo Life Sciences, Rome, Italy) was employed. The day of assay, the extracted vacuum-dried samples, which had been stored at −80 °C, were thawed at room temperature and suspended into 150 µL of Assay buffer, yielding an approximately 30–40 X concentrated solution. For the calibration curve, 7 dilutions of standard OT, ranging from 15.6 to 1000 pg/mL, were prepared from the OT stock solution (10,000 pg/mL). 

After incubation and washing procedures carried out with all the reagents and buffers provided by the kit, the microplate absorbance (Abs) was read at 405 nm, using the spectrophotometer Victor Wallac (Perkin Elmer, Milan, Italy). Color development, due to *p-*nitrophenol formation from *p-*nitrophenylphosphate and phosphatase alkaline-labeled OT, was inversely proportional to the sample content of unlabeled endogenous OT.

The calibration curve was calculated following the instructions provided by the kit and sample unknown OT amounts were interpolated as pg/mL, considering the concentration factor. The method’s sensitivity for OT measurement in plasma was 11/12 pg/mL.

The EIA kit was also used to validate the OT extraction procedure. Briefly, extraction recoveries of OT were appraised by comparing 405 nm Abs of OT standard solutions before and after Oasis C18 extraction. Oasis-C18 extracted plasma spiked with OT standards was compared, too. Valuable and reproducible recoveries resulted, accounting for 98–110%.

### 2.5. Cognitive and Neuropsychological Evaluation

A first battery of tests was used to investigate the psychocognitive abilities of the enrolled subjects. Subjects underwent the following tests: the MMSE for global cognitive state [30]; the MoCA [31]; the Frontal Assessment Battery (FAB) on mental flexibility, motor programming, sensitivity to interferences, inhibitory control, and environment autonomy [32]; and the Short-Term Intelligence Test [33] on intelligence and reading abilities.

A second series of evaluations consisted instead of social cognition tests using the following questionnaires: the “Faux-Pas Task” [34]; the perception of faces and emotions according to the KDEF [35]; the test of emotion attribution after a verbal trigger [36]; the empathy “Strange Stories” test [37]; and the Wilhelm Bush test [38]. All tests were performed by a skilled psychologist and a qualified neurologist of the Neurology Section of the Department of Clinical and Experimental Medicine of the University of Pisa.

All these cognitive and social cognition tests were applied twice, at baseline (T0) and 2 years after (T1), in HD patients.

### 2.6. Statistical Analysis

All statistical analyses were performed with the software IBM SPSS Statistics for Windows, Version 24.0. Armonk, NY: IBM Corp. using non-parametric statistical tests. The significance level in all statistical tests was set to 0.05. Comparisons between HD and controls were performed using the Mann Whitney U test for independent samples. Correlations were assessed using Spearman’s rank correlation coefficient. Descriptive statistics and calculation of the calibration curve for OT plasma levels (pg/mL) were carried out by means of the Graph-Pad Prism software (version 5, San Diego, CA, USA).

## 3. Results 

### 3.1. Baseline Analysis

At the baseline, the two populations of study did not differ in age (HD 65.4 ± 10.3; HC 61.1 ± 11.9, *p* = 0.36; Cohen’s d 0.39) and educational level (HD 8.6 ± 3.4; HC 8.2 ± 3.9, *p* = 0.75; Cohen’s d 0,13) (Table 1).

HD patients showed significantly lower performances at MMSE (*p* = 0.027; d 1.09), MoCa (*p* = 0.023; d 1.22) and FAB (*p* = 0.02; d 1.5). 

**Figure 1:**
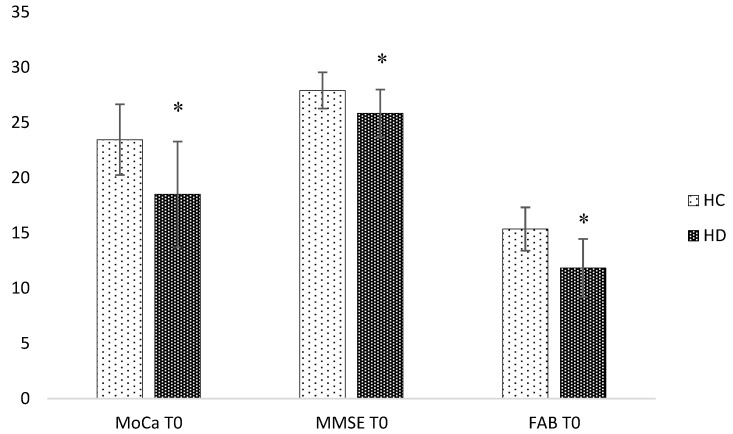
Differences between means in cognition at the baseline. HC: Healthy Controls. HD: Huntington’s Disease. MoCa: Montreal Cognitive Assessment; MMSE: Mini Mental State Examination; FAB: Frontal Assessment Battery. * *p* < 0.05. Error Bar ± 1 standard deviation.

The mean score for MMSE was HC 27.9 (DS 1.6), HD 25.8 (DS 2.2), respectively, whereas the MoCa score was HC 23.5 (± 3.2), HD 18.5 (± 4.8) (Figure 1).

**Figure 2:**
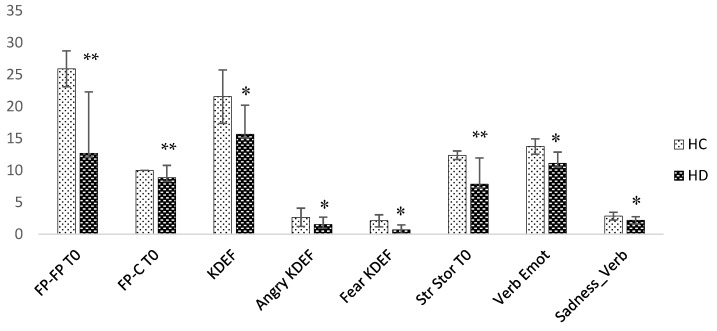
Differences in social cognition at the baseline. HC: Healthy Controls. HD: Huntington’s Disease. FP_FP: Faux-Pas_Faux Pas; FP_C: Faux-Pas_Control; KDEF: Karolinska Directed Emotional Faces; Angry_KDEF: Angry expression at Karolinska Directed. Emotional Faces; Fear_KDEF: Fear expression at Karolinska Directed Emotional Faces; Str_Stor: Strange Stories test; Verbal_Emotions: Verbal Emotions test; Sadness_Verb: Sadness at Verbal Emotions test. * *p* < 0.05, ** *p* < 0.01. Error Bar ± 1 standard deviation.

HD patients showed lower performances in the social cognition battery, in particular at the Faux-Pas test (subitems FP/control and FP/FP *p* = 0.01; respectively d 64.88 and 1.8), and the Strange stories test (*p* = 0.01; d 20.84). Significant difficulties in recognizing emotions from visual (*p* = 0.04; d 0.19) and verbal stimuli (*p* = 0.03; d 1.74) emerged, in particular for negative emotions, such as anger (Anger-KDEF *p* = 0.04; d 1.90), fear (Fear-KDEF *p* = 0.02; d 3.04), and sadness (Sad-Verb Emot *p* = 0.03; d 4.76) (Figure 2).

**Figure 3:**
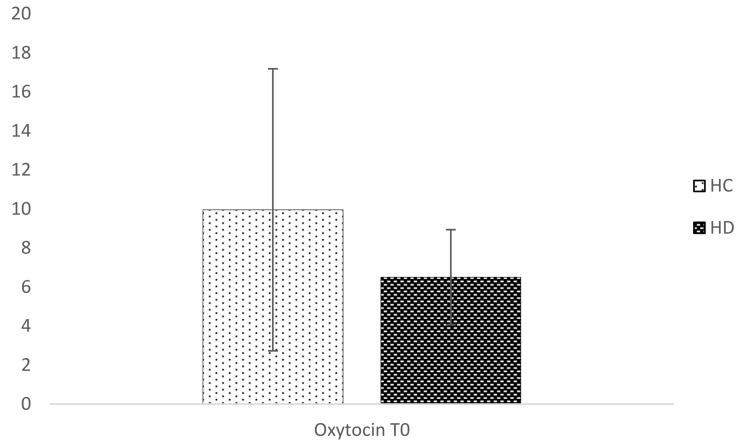
Differences in plasma Oxytocin at the baseline. HC: Healthy Controls. HD: Huntington’s Disease. *p* = 0.566. Error Bar ± 1 standard deviation.

OT plasma levels did not differ in the two populations, despite a trend for lower values in the HD group (average 6.5 ± 2.4 pg/mL versus 9.9 ± 7.2 pg/mL; Glass delta 0.44) at the baseline (Figure 3).

No correlation between OT levels and neuropsychological scores was observed in HD at T0, although patients displayed a higher baseline.

### 3.2. Longitudinal Analysis

The Wilcoxon analysis did not show any differences in MMSE and MoCa at T0 and T1, respectively (*p* = 0.26 and *p* = 0.14). 

OT levels had better performances at the Faux-Pas test (Neutral\FP *p* = 0.03) after two years (T1).

Moreover, the follow-up analysis evidenced a positive correlation between MoCa scores at the baseline and Strange Stories (*p* = 0.001), KDEF, in particular disgust (*p* = 0.003), as well as MMSE at the baseline and Strange Stories (*p* = 0.01), KDEF, in particular neutral (*p* = 0.04) (Table 2). 

## 4. Discussion

The present study confirmed previous results of literature, reporting impairment of cognitive and executive functions in HD patients compared to controls, as shown by the MMSE, MoCa and FAB scores. Moreover, patients showed an extensive impairment of social cognition, in particular at the Faux-Pas task (Faux-Pas_Faux Pas and Faux-Pas_Control (FP/FP and FP/C)), Strange Stories, with reduced ability to recognize fear and anger based on facial expression, and finally impairment in perception of verbal emotions, sadness in particular.

Although the scales used were different from the scales reported in previous studies, our data supported impairment of ToM during the course of the disease in HD patients. The cognitive as well as the affective component of the ToM seemed to be involved.

This finding is in line with a study conducted on 18 HD patients in the initial phase of the disease and 18 healthy controls, in which the ToM assessment included two tests: A first test where the subject was required to attribute the intentions of subjects depicted in some stories, followed by logical questions of control of understanding of the story itself (cognitive ToM), and a second represented by a version of the known Reading the Mind in the Eyes (affective ToM). The two components were equally involved in the group of patients [39].

The first data regarding emotion recognition date back to 1996, when Sprengelmeyer, by using “Ekman and Friesen series”, evidenced the inability of HD patients to recognize disgust from facial expressions [40]. During the last 20 years, lots of studies have supported these data even for auditory, olfactory and gustatory stimuli [41,42]

Although previous studies differ greatly in the type of scales used, a review made by Henley et al. in 2012 showed difficulties in recognizing expressions, particularly with regard to fear and disgust, in HD and pre-symptomatic HD patients [4].

We failed to detect a significant reduction of OT in HD patients; however, a trend for lower plasma levels was observed. To our knowledge, this is the first study analyzing OT plasma levels.

Hypothalamic and OT involvement seems to be independent from the HD stage, as suggested by VBM magnetic resonance studies that have shown a reduction of the hypothalamus in patients even 10 years before symptom onset [43], and by anatomopathological reports [44]. Gabery et al. evidenced the loss of OT neurons in HD patient who suffered a cardiac arrest a few months after diagnosis. The patient presented an anatomopathological Vonsattel grade 0, and no atrophy nor pathology at the macroscopic evaluation of the hypothalamus, but the immunohystochemical study showed a reduction of 45% and 24% in the number of neurons of OT and Vasopressin, respectively [44].

Despite the small sample size of our stage-II HD patients, we could report a trend for lower OT levels in plasma than HC. It is also noteworthy that OT plasma levels in HD patients were also much less variable in respect to controls. It is thus tempting to hypothesize that bloodstream oxytocinergic responses are impaired in HD. 

The plasma concentration of OT is extremely low, both in non-pregnant women and in men. The hormone circulates in the plasma as a monomer not bound to proteins. It has a half-life of about 5 min and is catabolized in the liver and kidney through a deacetylation process [45]. The half-life of OT in the central nervous system is 28 min and presents a circadian peak around midday which is not found in plasma [46,47]. These data suggest that the peripheral and central OT levels could have different and independent regulation mechanisms. In support of this hypothesis, a more recent work has shown that OT levels are higher in the CNS than in plasma and that the two values are not correlated with each other [48]. On the other hand, other studies have found significant correlations between OT plasma levels and depressive, autism-spectrum, or psychotic symptoms [49]. Peripheral OT would thus mirror a complex neuroendocrine pattern rather than simply the hypothalamic release.

The follow-up analysis showed a correlation between social cognition performances and baseline OT plasma level, and cognitive performances. The correlation between basal OT values and follow-up social cognition performances might suggest the potential role of OT in mediating social cognition, with a predictive value in long-term periods. OT has been implicated in many psychiatric conditions, such as social phobia, post-traumatic stress disorder, obsessive-compulsive disorder, and depression. Several studies in the literature have correlated changes in the plasma levels of OT with altered affective behaviors in the context of neuropsychiatric pathologies [50,51], or with the state of anxiety in healthy people [52].

Intranasal administration of OT in HD patients has been shown to improve emotion recognition, particularly regarding angry faces [53]. To support the role of OT in social cognition and psychiatric disorders, Boccia et al. analyzed the distribution of the OT receptor in the human brain. By using monoclonal antibodies, 2F8 directs to the uterine OT receptor the evidenced positive reaction in discrete cell bodies and/or fibers in the central and basolateral regions of the amygdala, medial preoptic area (MPOA), anterior and ventromedial hypothalamus, olfactory nucleus, vertical limb of the diagonal band, ventrolateral septum, anterior cingulate, and hypoglossal and solitary nuclei [54]. One central mechanism of oxytocinergic action is likely to be mediated by the amygdala. Kirsch et al. (2005) conducted a functional Magnetic Resonance study showing that intranasal OT decreased amygdala activation (after the subjects had been challenged with fear-provoking pictures of faces and aversive scenes) [55].

Our finding of a correlation between cognitive performance and social cognition confirms previous reports in which this correlation was stronger with numbers of perseverative errors, executive planning skills and verbal fluency [56]. The role of executive functions in social cognition has recently been confirmed in a larger study conducted on 50 manifest HD patients [57]. Because these executive functions are better evaluated by the MoCa test than MMSE, this is a possible explanation for our finding of a stronger correlation between MoCa and ToM performances.

The present study, though limited by the small sample size and the biological sample analyzed, supports a correlation between OT and HD social cognition, evidencing a possible pathophysiological role of OT in the disease and the therapeutic usefulness of this hormone. To confirm this hypothesis and apply predictive multivariate statistical models, a wider number of subjects, even in other biological matrices, such as, in particular, the cerebrospinal fluid, should be investigated. Moreover, results obtained in this work suggest the importance of scales such as MoCa in evaluating patients, especially due to their capacity to explore executive functions more involved in HD cognition.

## Figures and Tables

**Table 1:** Demographic characteristics of patients and controls.

Demographic Characteristics	HD(*n*13)	HC(*n*11)
Sex	4F,9M	3F,8M
Age(years)	61.08 ± 11.90 (45–78 years)	65.45 ± 10.34 (45–78 years)
Educational level(years)	8.66 ± 3.44 (5–13 years)	8.18 ± 3.91 (5–13 years)

HD: Huntington’s Disease. Results are shown as mean (± standard deviation). HC: Healthy Controls.

**Table 2:** Correlations at the follow-up analysis in HD population.

	FP/N_T1	Str-Stor_T1	KDEF_Tot_T1	Disg_KDED_T1	Neutr_KDEF_T1
OT_T0	*p* = 0.03 *	-	-	-	-
MOCa_T0	-	*p* = 0.001 **	*p* = 0.007 **	*p* = 0.003 **	
MMSE_T0	-	*p* = 0.01 *	*p* = 0.03 *	-	*p* = 0.04 *

HD: Huntington’s Disease. FP_N: Faux-Pas_Neutral; Str_Stor: Strange Stories test; KDEF: Karolinska Directed Emotional Faces; Disg_KDEF: Disgust expression at Karolinska Directed. Emotional Faces; Neutr_KDEF: Neutral expression at Karolinska Directed Emotional Faces. * *p* < 0.05, ** *p* < 0.01.
